# Factors Influencing Resection Time in Endoscopic Submucosal Dissection for Rectal Neuroendocrine Tumors

**DOI:** 10.1002/deo2.70199

**Published:** 2025-09-02

**Authors:** Shinya Nakatani, Junnosuke Hayasaka, Tsuyoshi ishii, Minoru Oda, Yusuke Kawai, Hiroshi Yamato, Yorinari Ochiai, Yugo Suzuki, Yutaka Mitsunaga, Hiroyuki Odagiri, Akira Matsui, Yasuro Miura, Shu Hoteya

**Affiliations:** ^1^ Department of Gastroenterology Toranomon Hospital Tokyo Japan; ^2^ Department of Pathology Toranomon Hospital Tokyo Japan

**Keywords:** endoscopic submucosal dissection, neuroendocrine tumor, rectum, procedure time, traction device

## Abstract

**Objectives:**

The usefulness of endoscopic submucosal dissection (ESD) for rectal neuroendocrine tumors (NETs) is well established. However, factors influencing resection time remain unclear. This study aimed to identify these factors during ESD for rectal NETs.

**Methods:**

This retrospective study included 194 rectal NET lesions that were treated with ESD at our institution between March 2011 and July 2024. Potential factors influencing resection time—including age, sex, operator experience (non‐expert endoscopist: <50 colorectal ESD cases), sodium hyaluronate use, traction device (TD) use, tumor location, lesion size, and specimen area—were analyzed using multiple regression analysis.

**Results:**

The median resection time was 30 min (interquartile range [IQR]: 20–43 min). Non‐expert endoscopists performed 53% of the procedures. The median specimen area was 302 mm^2^ (IQR: 233–393 mm^2^). Resection time was significantly longer when procedures were performed by non‐experts (β = 8.66; 95% confidence interval [CI]: 4.46–12.86; *p* <0.001), when the tumor was located in the upper rectum (Rs) compared to the lower rectum (Rb) (β = 20.96; 95% CI: 7.82–34.1; *p* = 0.002), and with increasing specimen area (β = 0.04; 95% CI: 0.027–0.06; *p* <0.001). Conversely, TD use significantly shortened resection time (β = ‐5.90; 95% CI: ‐11.37 to ‐0.43; *p* = 0.036).

**Conclusions:**

Traction device use during ESD for rectal NETs is associated with shorter resection time; whereas, procedures performed by non‐experts, tumors located in the Rs, and larger specimen areas were associated with longer resection time.

## 1 Introduction

Rectal neuroendocrine tumors (NETs) are submucosal (SM) tumors that originate from neuroendocrine cells located in the lower section of the rectal crypts [1]. The incidence of rectal NETs has increased fivefold—from 1.09 per 100,000 individuals in 1973 to 5.25 per 100,000 in 2004—likely due to the introduction of the World Health Organization classification, widespread use of colonoscopy, and greater clinical awareness [2, 3, 4, 5, 6, 7].

Rectal NETs often infiltrate the SM layer regardless of tumor diameter [8]. Small rectal NETs have a low risk of metastasis and are therefore considered suitable for endoscopic treatment [9].

Various endoscopic resection techniques have been developed for rectal NETs, including endoscopic mucosal resection (EMR), cap‐assisted EMR (EMR‐C) [10, 11], endoscopic SM resection with a ligation device (ESMR‐L) [12, 13], and endoscopic SM dissection (ESD) [14, 15]. Among these, ESMR‐L and ESD are commonly used because of their higher R0 resection rates compared to those of EMR [16].

However, ESD for rectal NETs differs from that for typical colorectal epithelial tumors due to the small tumor size and high rate of SM infiltration. In Japan, endoscopic treatment is indicated for small (<1 cm) non‐invasive rectal NETs [17]. In contrast, ESD for epithelial tumors is generally performed for larger lesions suspected to be intramucosal carcinoma, and is also applied to lesions with SM invasion or fibrosis [18].

Factors influencing resection time during ESD for rectal epithelial tumors include involvement of the dentate line, lesion diameter >50 mm, circumferential involvement >2/3, and T1b depth of invasion—all of which are associated with longer procedure time [19]. In our previous study, although not a primary endpoint, the use of traction devices (TDs) was associated with shorter resection time during ESD for rectal NETs [20].

Shortening resection time is relevant, as prolonged procedures may increase operator fatigue and procedural risk [21].

However, factors affecting resection time in ESD for rectal NETs remain unclear. Therefore, this study aimed to identify these factors.

## 2 Methods

### 2.1 Patients

**FIGURE 1:**
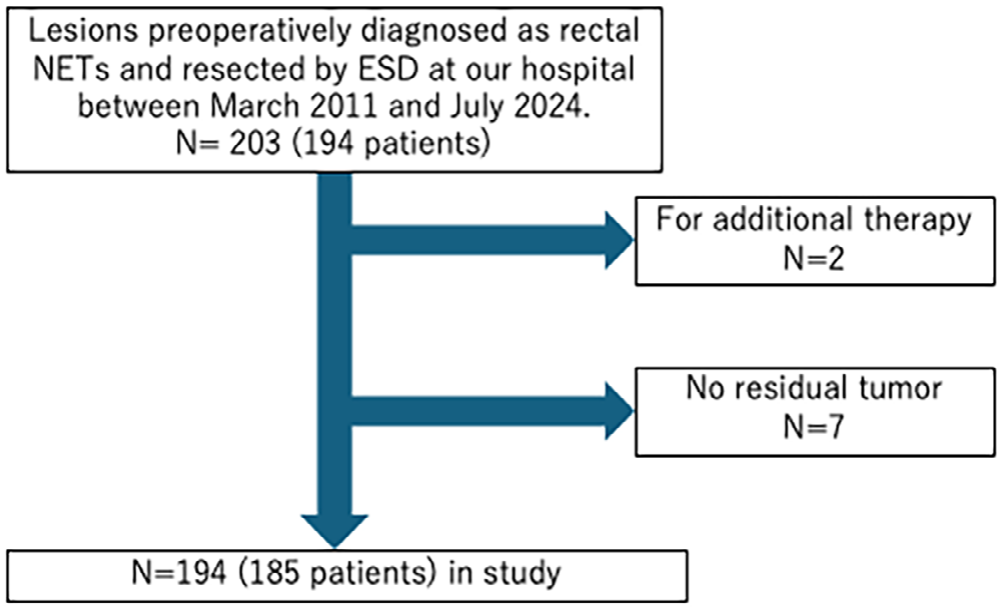
Flow diagram of patient selection for the study. ESD, endoscopic submucosal dissection; NET, neuroendocrine tumor.

This single‐center, retrospective study included 203 lesions treated with ESD for rectal NETs at our institution between March 2011 and July 2024. Seven lesions that were initially diagnosed as NETs by biopsy but were later confirmed as non‐NETs following ESD, as well as two lesions that underwent ESD as additional therapy, were excluded. Ultimately, 194 lesions from 185 patients were included in this study (Figure 1). Written informed consent was obtained from all patients, and the study was approved by the Institutional Review Board of Toranomon Hospital (approval number: 2471) in accordance with the Declaration of Helsinki.

Patient records were reviewed to collect data on the following variables: age, sex, operator experience, sodium hyaluronate use, TD use, resection time, lesion location, degree of SM fibrosis, lesion size, specimen area, NET grade, presence of lymphovascular invasion, and resection margins. The primary endpoint of this study was resection time, selected to identify factors associated with procedural difficulty and technical efficiency. Resection time is widely accepted as a surrogate marker for these aspects [22, 23].

### 2.2 Definitions

Resection time was defined as the duration from the initiation of mucosal incision to the completion of en bloc resection. In TD cases, preparation and attachment times were included. Operators with experience in fewer than 50 colorectal ESD procedures were defined as non‐expert endoscopists, whereas those with experience in 50 or more were defined as expert endoscopists. The area of the resected specimen was calculated using the formula for the area of an ellipse: area = (long axis × short axis × π)/4, where the long and short axes were measured from the formalin‐fixed specimen. The TD group included procedures using the Multi‐Loop TD (MLTD; Boston Scientific, Marlborough, USA), S‐O Clip (Zeon Medical Inc., Tokyo, Japan), or the thread‐traction method. Lesion locations were categorized as lower rectum (Rb), middle rectum (Ra), or upper rectum (Rs) segments. Submucosal fibrosis was classified as F0 (none), F1 (loose spiderweb‐like fibrosis), or F2 (dense, white fibrosis) [24, 25].

R0 resection was defined as en bloc resection with histologically tumor‐free lateral and vertical margins, based on pathological assessment of the formalin‐fixed specimen.

ESD Procedure The endoscope models used (GIF‐Q260J, GIF‐2TQ260M, PCF‐Q260J, PCF‐H290TI, and GIF‐H290T; Olympus, Tokyo, Japan) and distal attachments were selected at the discretion of the operator. Submucosal injection was performed using glycerol with indigo carmine, with sodium hyaluronate added based on operator preference. A Dual‐J Knife (1.5 mm; Olympus) was used for dissection. Markings were made approximately 5 mm away from the lesion, followed by a mucosal incision.

**FIGURE 2:**
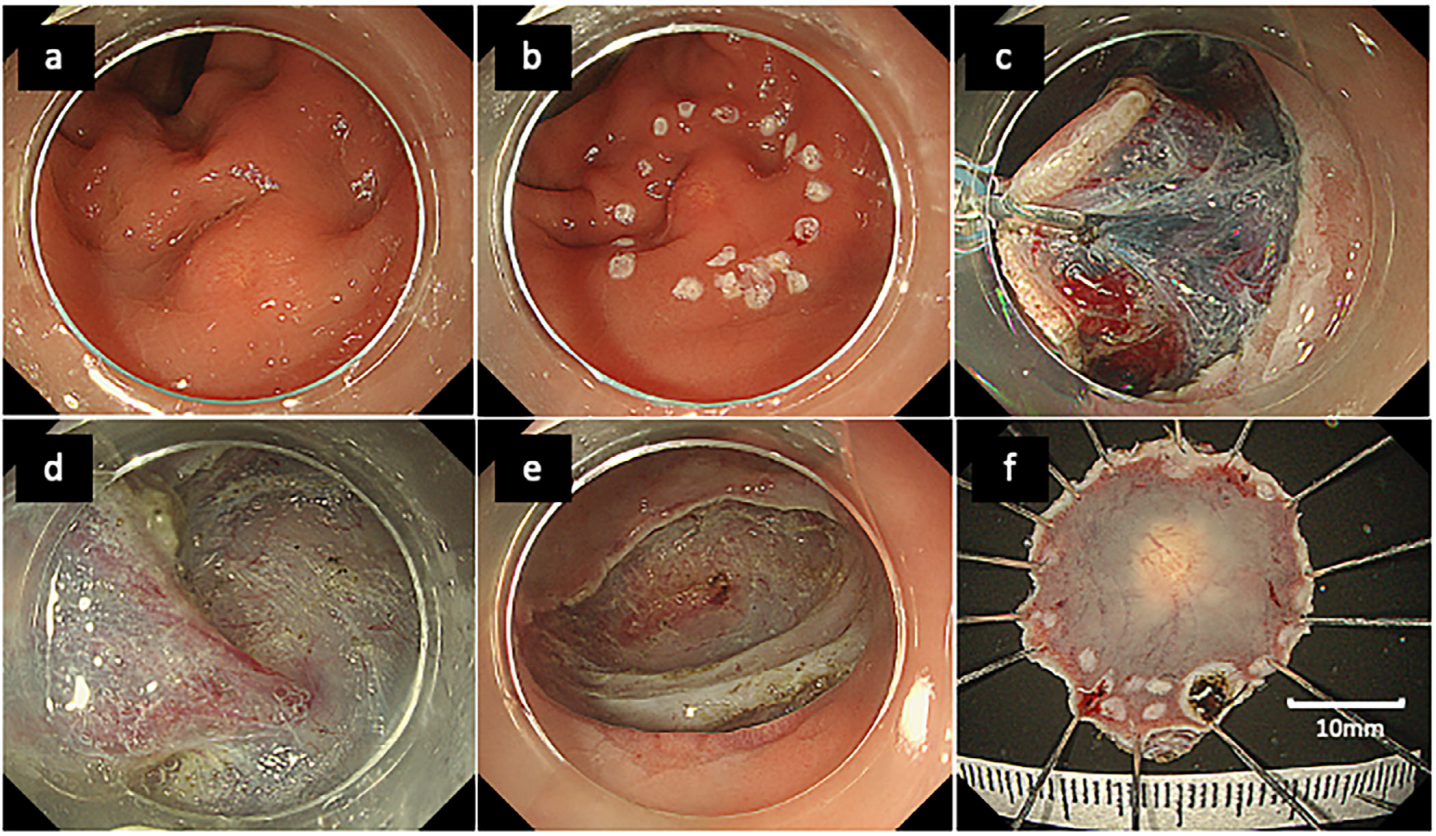
Step‐by‐step endoscopic submucosal dissection (ESD) of a rectal neuroendocrine tumor using a multi‐loop traction device. (a) Endoscopic view of a 7‐mm neuroendocrine tumor in the Ra segment of the rectum. (b) Marking dots placed circumferentially around the lesion. (c) Full circumferential mucosal incision prior to dissection, followed by the application of traction using a multi‐loop traction device. (d) Dissection phase assisted by the multi‐loop traction device. (e) Post‐ESD ulcer bed observed after complete resection. (f) Resected specimen after ESD. Ra, middle rectum.

In standard ESD, the incision was initiated on the oral or anal side, followed by dissection in the same direction. A full circumferential incision was completed after >50% of the dissection had been performed. Traction device (TD) use was retrospectively classified into two categories based on the timing and intent of application. In planned TD cases, traction was applied preemptively before the start of SM dissection, typically after completing a full circumferential incision. This approach was selected according to lesion characteristics or operator preference (Figure 2). In contrast, rescue TD use referred to intra‐procedural application of a TD in response to technical difficulties.

### 2.3 Assessment of Postoperative Complications

Postoperative complications, including delayed bleeding, perforation, and post‐ESD coagulation syndrome (PECS), were retrospectively identified. Delayed bleeding was defined as onset within 30 days after ESD of overt bleeding—such as hematemesis, hematochezia, or melena—or a hemoglobin decrease ≥ 2 g/dL—combined with the need for endoscopic/surgical intervention, blood transfusion, or prolonged hospitalization. Intraoperative perforation was defined as an endoscopically visible hole in the colorectal wall exposing peritoneal fat or abdominal organs during the procedure, or post‐ESD detection of free air or radiologic perforation signs accompanied by clinical symptoms such as peritonitis or abdominal pain PECS was defined as localized abdominal pain and fever (≥37.6 °C), leukocytosis (≥10,000/µL), or elevated CRP (≥0.5 mg/dL) occurring ≥6 h after ESD [26].

### 2.4 Statistical Analysis

Continuous variables are presented as medians (interquartile range [IQR]), and categorical variables as percentages. Multiple regression analysis was performed to identify factors influencing resection time. Candidate variables included age, sex, operator experience, sodium hyaluronate use, TD use, lesion location, lesion size, and specimen area. Depth of invasion and degree of fibrosis were not included in the analysis, as all lesions exhibited SM invasion and were classified as F0 fibrosis. Prior to conducting multivariate regression analysis, standard model assumptions—linearity, normality of residuals, homoscedasticity, and absence of multicollinearity—were assessed. No significant violations were identified. As a subgroup analysis, to assess the true procedural impact of TD when used in a planned fashion, a subgroup analysis was performed excluding cases in which TD was introduced as a rescue tool during technically difficult procedures.

To assess the clinical significance of resection time, patients were stratified into two groups: ≤30 and >30 min (based on the median resection time). Complication rates were compared between the two groups using Fisher's exact test. A composite outcome was also created, defined as the occurrence of any complication (delayed bleeding, perforation, or PECS).　A *p*‐value < 0.05 was considered statistically significant. All analyses were performed using R software, version 4.1.3 (The R Foundation for Statistical Computing, Vienna, Austria).

## 3 Results

The baseline characteristics of the patients are summarized in Table 1. The median patient age was 56 years; 62% of the patients were male. Non‐expert endoscopists performed 53% of the procedures. The median resection time was 30 min. The Rb segment was the most common lesion location, accounting for 74% of cases. The median lesion size was 5 mm (IQR: 4–8 mm). The median specimen area was 303 mm^2^ (IQR: 233–393 mm^2^).

**TABLE 1:** Baseline characteristics of lesions treated with endoscopic submucosal dissection (ESD) for rectal neuroendocrine tumors (NETs) (*N* = 194).

Charcteristics	*n* = 194
Age: Median (IQR), years	56 (24–82)
Sex: Male	121 (62.3%)
Hyaluronic acid	38 (19.6%)
Non‐Expert	91 (46.9%)
Tumor size: Median (IQR), mm	5 (4‐8)
Specimen area: Median (Range), mm^2^	302.7 (55–1074)
Location:	
Rb	146 (75.3%)
Ra	43 (22.2%)
Rs	5 (2.5%)
Traction Device	39 (20.1%)
Multi‐Loop Traction Device	32
Theread‐Traction	6
S‐O Clip	1
World Health Organization Classification: NET G1/G2/G3	
G1	188 (96.9%)
G2	6 (3.1%)
G3	0 (0%)
Lymphatic invasion	27 (13.9%)
Venous invasion	33 (17.0%)
Horizontal margin negative	192 (99.0%)
Vertical margin negative	188 (96.9%)
R0 resection	187 (96.4%)
Complication:	
Delayed bleeding	4 (2.1%)
Intraoperative perforation	1 (0.5%)
Post‐ESD coagulation syndrome	4 (2.1%)
Postprocedural stenosis	0 (0%)
Recurrence	0 (0%)

ESD, endoscopic submucosal dissection; IQR, interquartile range; NET, neuroendocrine tumor; WHO, World Health Organization.

^†^ Non‐expert endoscopists were defined as those with experience in <50 colorectal ESD procedures.

^‡^ Rb, rectum below the peritoneal reflection; Ra, rectum above the peritoneal reflection; and Rs, rectosigmoid.

R0 resection was achieved in 96.4% of cases, with horizontal and vertical margin negativity rates also reaching almost 97%, indicating a high rate of curative resection.

Postoperative complications were observed in eight patients (4.1%) in the overall cohort. Specifically, delayed bleeding occurred in four patients (2.1%), intraoperative perforation in one patient (0.5%), and post‐ESD coagulation syndrome (PECS) in three patients (1.5%).

Multiple regression analysis identified non‐expert endoscopist status (β = 8.66; 95% confidence interval [CI]: 4.46–12.86; *p* <0.001), Rs location (β = 20.96; 95% CI: 7.82–34.1; *p* = 0.002), and specimen area (β = 0.04; 95% CI: 0.027–0.06; *p* <0.001) as factors significantly associated with prolonged resection time. TD use was associated with shorter resection time (β = ‐5.90, 95% CI: ‐11.37 to ‐0.43; *p* = 0.036) (Table 2).

**TABLE 2:** Clinical factors associated with resection time in endoscopic submucosal dissection for rectal neuroendocrine tumors, based on multiple regression analysis.

Variables	Β‐coefficient	95% Confidence interval	*p*‐Value
Age (years)	−0.03	−0.2 to 0.14	0.75
Sex, Male	2.47	−3.4 to 0.98	0.27
Sodium hyaluronate use	5.06	−0.19 to 10.31	0.061
Non‐expert endoscopist^a^	8.66	4.46 to 12.86	<0.001^***^
Tumor size (mm)	−0.01	−0.92 to 0.90	0.98
Specimen area (mm^2^)	0.04	0.03 to 0.06	<0.001^***^
Tumor location^b^			
−Rb (reference)	—	—	
−Ra	4.19	−0.77 to 9.16	0.100
−Rs	20.96	7.82 to 34.11	0.002^**^
Traction device use	−5.90	−11.37 to ‐0.43	0.036^c^

^a^ Non‐expert endoscopists were defined as those with experience in <50 colorectal ESD procedures.

^b^ Rb, rectum below the peritoneal reflection; Ra, rectum above the peritoneal reflection; and Rs, rectosigmoid.

*p* < 0.05 was considered statistically significant.

^c^ Fisher's exact test was used for categorical comparisons.

A total of 36 cases with planned TD use were included in this analysis (rescue TD 3 cases excluded). In this subgroup, multivariable linear regression identified several factors independently associated with resection time (Table 3).

**TABLE 3:** Factors associated with resection time in cases with planned traction device use.

Variables	Β‐coefficient	95% Confidence Interval	*p*‐Value
Age (years)	−0.02	−0.19 to 0.15	0.82
Sex, Male	2.47	−1.65 to 7.12	0.22
Sodium hyaluronate use	5.36	−0.08 to 10.64	0.048
Non‐expert endoscopist^†^	8.31	4.06 to 12.6	<0.001^***^
Tumor size (mm)	−0.036	−0.95 to 0.88	0.94
Specimen area (mm^2^)	0.04	0.03 to 0.06	<0.001
Tumor location^b^			
−Rb (reference)	—	—	
−Ra	4.25	−0.74 to 9.16	0097
−Rs	20.96	1.42 to 30.69	0.003
Traction device use	−6.65	−12.3 to ‐0.4	0.036^a^

*p* < 0.05 was considered statistically significant.

^a^ Fisher's exact test was used for categorical comparisons.

^b^ Rb, rectum below the peritoneal reflection; Ra, rectum above the peritoneal reflection; and Rs, rectosigmoid.

A planned TD use remained significantly associated with shorter resection time (β = –6.65, 95% CI: –12.32 to –0.4, p = 0.036), and the effect size was even greater than that observed in the primary analysis (β = –5.39).

This finding suggests that the planned use of TDs may be particularly effective in shortening procedure time.

Postoperative complications were stratified by resection time (≤30 min vs. >30 min based on the median), and the results are summarized in Table 4.

**TABLE 4:** Association of resection time with oncological outcomes and complications in endoscopic submucosal dissection for rectal neuroendocrine tumors.

Variable	≤30 min (*n* = 103)	>30 min (*n* = 91)	*p*‐Value
**Oncological outcomes**			
Horizontal margin Negative	102 (99.0)	90 (98.9)	1.00
Vertical margin Negative	101 (98.1)	87 (95.6)	0.42
R0 Resection	100 (97.1)	87 (95.6)	0.71
Recurrence	0 (0.0)	0 (0.0)	—
**Complications**			
Delayed bleeding (%)	0 (0.0)	4 (4.4)	0.047
Perforation (%)	0 (0.0)	1 (1.1)	0.47
PECS (%)	0 (0.0)	3 (3.3)	0.101
Delayed bleeding, perforation, and PECS (%)	0 (0.0)	8 (8.8)	0.002
Postprocedural stenosis	0 (0.0)	0 (0.0)	—

PECS, Post‐endoscopic submucosal dissection coagulation syndrome.

No complications were observed in the ≤30‐min group. In contrast, in the >30‐min group, delayed bleeding occurred in four cases (4.4%) and perforation in one case (1.1%), and PECS in three patients (3.3%).

In the group ≤30‐min group, the rate of delayed bleeding was significantly lower (*p* = 0.047), while perforation and PECS were lower but did not reach statistical significance (*p* = 0.47 and 0.101). The composite complication rate (delayed bleeding, perforation, and PECS) was significantly higher in the >30‐min group (8.8%) compared to the ≤30‐min group (0%) (*p* = 0.002).

The R0 resection rate was slightly higher in the ≤30‐min group than in the >30‐min group (97.1% vs. 95.6%), although the difference was not statistically significant (*p* = 0.71).

Horizontal and vertical margin negativity were also comparable between the groups (horizontal: 99.0% vs. 98.9%, *p* = 1.000; vertical: 98.1% vs. 95.6%, *p* = 0.42).

## 4 Discussion

This retrospective study identified key factors influencing resection time in ESD for rectal NETs. To our knowledge, this is the first study to specifically evaluate such factors. Our study demonstrated that longer resection times were associated with non‐expert endoscopists, Rs‐located lesions, and larger specimen areas, whereas shorter resection times were associated with the use of TDs.

Recent case reports have demonstrated the utility of TDs in rectal NET ESD [27, 28]. Our previous study suggested that TD use may reduce resection time [20]. However, a multicenter randomized controlled trial (RCT) reported no reduction in resection time with TDs for colorectal epithelial tumors [29]. Of note, the RCT included larger lesions (>20 mm) with minimal SM invasion (6%), unlike our study population of small, submucosa‐invading NETs. Differences in tumor pathology and characteristics may explain the discrepancy. Another multicenter RCT showed a trend toward shorter resection time, such as procedures performed by non‐expert operators, suggesting that TDs may offer procedural advantages under selected conditions [30].

Subgroup analysis of TD use further supports its procedural benefit.

To minimize potential reverse causality, we conducted a subgroup analysis excluding the rescue method. In this analysis, planned TD use was associated with a greater reduction in resection time (β = ‐6.65) compared to the overall cohort (β = ‐5.90), emphasizing the importance of preemptive traction.

These findings demonstrate the robustness of the association between planned TD use and reduced resection time, supporting its utility in maximizing procedural efficiency during rectal NET ESD.

Lesions in the Rs segment present greater technical challenges due to tangential access, narrow lumen, and distinct anatomical features compared with Rb lesions. These complexities may necessitate more attention from the surgeon and longer resection time owing to the risk of perforation. Our finding that larger specimen areas correlated with longer resection time aligns with reports on epithelial tumors [31]. It is noteworthy that lesion size itself did not significantly affect resection time, possibly because the tumors in this cohort were relatively small (median diameter: 5 mm; IQR 4–8 mm^2^). The influence of specimen area—rather than lesion size—on procedure time may reflect how the surgeon marked and determined incision margins relative to lesion size.

Non‐expert endoscopists took longer, likely due to challenges in flap creation and navigating dissection depth in small, submucosa‐invading NETs. Therefore, TDs may help overcome these challenges by eliminating flap creation. Although this observation stems from a subgroup analysis of an RCT, it suggests that TD use in real‐world colorectal ESD procedures could shorten resection time for non‐expert endoscopists [29].

Based on our findings, TDs may be recommended for Rs‐located lesions or non‐expert endoscopists, as they may reduce specimen area, eliminate flap creation, and shorten procedure time.

The present study highlights the importance of identifying modifiable factors that influence procedural efficiency in ESD for rectal NETs. Addressing modifiable factors can improve daily practice.

On the other hand, the impact of the identified factors on resection time was relatively modest. For instance, non‐expert endoscopist status was significantly associated with prolonged resection time; however, the mean difference was only approximately 8 min.

However, even an 8‐min increase corresponds to a 24% longer resection time, which can accumulate and potentially reduce procedural efficiency.

However, in low‐volume centers with less‐experienced operators, this time gap could be substantially greater. It is important that our study was able to demonstrate that, even in such settings, the use of TDs can be expected to improve procedural efficiency.

Our analysis revealed a significant association between prolonged resection time and the occurrence of postoperative complications in rectal ESD for NETs. Specifically, patients in the >30‐min group demonstrated significantly higher rates of delayed bleeding (4.4% vs. 0%, *p* = 0.047), as well as a higher cumulative incidence of bleeding, perforation, and PECS (8.8% vs. 0%, *p* = 0.002). These findings suggest that although prolonged procedural duration did not compromise R0 resection or margin negativity (with only a slight, non‐significant decrease), it was associated with a higher incidence of adverse events. Operator fatigue, tissue exposure time, and cumulative thermal damage may contribute to these risks [30]. Therefore, minimizing resection time may be clinically important not only for procedural efficiency but also for improving patient safety.

Comparison with EMR and ESMR‐L contextualizes ESD's clinical value for rectal NETs. While modified EMR techniques, such as EMR‐C or ESMR‐L, are widely used for small rectal NETs due to their technical simplicity, their procedural time is significantly shorter than that of ESD, typically 5–6 min vs. 20–30 min for ESD [31, 32].

Despite this time advantage, recent studies have shown comparable safety profiles between these methods. The overall adverse event rates—including delayed bleeding and perforation—remain below 5% for both EMR/ESMR‐L and ESD [33], suggesting longer procedural duration does not necessarily increase risk when performed by experienced operators.

In terms of curability, both EMR‐based techniques and ESD demonstrate R0 resection rates exceeding 90% for tumors <10 mm. However, ESD offers superior control of both horizontal and vertical margins, especially in lesions with SM fibrosis, poorly defined borders, or proximity to the dentate line [34, 35, 36].

Therefore, while EMR and ESMR‐L may be appropriate for routine, well‐demarcated, sub‐centimeter tumors, ESD should be considered when margin control is critical or lesion characteristics are technically demanding. ESD shows advantages in SM fibrosis, poorly demarcated borders, or lesions ≥8 mm [35, 37, 38]. These factors are known to reduce the efficacy or feasibility of modified EMR techniques such as EMR‐C or ESMR‐L. In this context, ESD remains a specialized but essential technique that ensures oncologic reliability, even when complexity increases [35, 37, 38].

This study has several limitations. First, it was a single‐center, retrospective analysis, introducing potential selection bias. Second, procedural details such as the use of hyaluronic acid and the choice of endoscope model were determined by each endoscopist's discretion and were not standardized. Third, the impact of fibrosis and depth could not be assessed due to their uniformity. Fourth, the long inclusion period (2011–2024) may have introduced chronological bias due to advances in endoscopic devices and techniques over time. Finally, TDs were sometimes used as rescue devices. To address this, we performed a subgroup analysis limited to cases with planned TD use. In this subgroup, planned TD use remained significantly associated with shorter resection time, suggesting that intentional implementation of TDs may contribute to procedural efficiency independent of case complexity.

## 5 Conclusion

In ESD for rectal NETs, operator experience, lesion location (Rs), specimen area, and TD use significantly influenced resection time. For non‐expert endoscopists or lesions located in the Rs, the use of TDs and efforts to minimize specimen area may be considered to potentially improve procedural efficiency. Such strategies may reduce procedure duration and help lower adverse event risk without compromising curability.

We are grateful to the physicians in the Department of Gastroenterology and Pathology at Toranomon Hospital for their valuable support in preparing the manuscript.

## Conflicts of Interest

The authors declare no conflicts of interest.

## Ethics Statement

Written informed consent was obtained from all patients, and the study was approved by the Institutional Review Board of Toranomon Hospital (approval number: 2471) in accordance with the ethical standards of the 1964 Declaration of Helsinki and its later amendments.

## Clinical Trial Registration

N/A

## References

[1] J. I. Gordon and M. L. Hermiston , “Differentiation and Self‐renewal in the Mouse Gastrointestinal Epithelium,” Current Opinion in Cell Biology 6 (1994): 795–803. 7880525 10.1016/0955-0674(94)90047-7

[2] J. C. Yao , M. Hassan , A. Phan , et al., “One Hundred Years After “Carcinoid”: Epidemiology of and Prognostic Factors for Neuroendocrine Tumors in 35,825 Cases in the United States,” Journal of Clinical Oncology 26 (2008): 3063–3072. 18565894 10.1200/JCO.2007.15.4377

[3] M. Y. Cho , J. M. Kim , J. H. Sohn , et al., “Current Trends of the Incidence and Pathological Diagnosis of Gastroenteropancreatic Neuroendocrine Tumors (GEP‐NETs) in Korea 2000–2009: Multicenter Study,” Cancer research and treatment: official journal of Korean Cancer Association 44 (2012): 157–165. 10.4143/crt.2012.44.3.157 PMC3467418 23091441

[4] H. J. Tsai , C. C. Wu , C. R. Tsai , et al., “The Epidemiology of Neuroendocrine Tumors in Taiwan: A Nationwide Cancer Registry‐based Study,” PLoS ONE 8 (2013): e62487. 23614051 10.1371/journal.pone.0062487 PMC3632554

[5] M. Fraenkel , M. Kim , A. Faggiano , et al., “Incidence of Gastroenteropancreatic Neuroendocrine Tumours: A Systematic Review of the Literature,” Endocrine‐Related Cancer 21 (2014): R153–R163. 24322304 10.1530/ERC-13-0125

[6] E. Leoncini , P. Boffetta , M. Shafir , et al., “Increased Incidence Trend of Low‐grade and High‐grade Neuroendocrine Neoplasms,” Endocrine 58 (2017): 368–379. 28303513 10.1007/s12020-017-1273-x PMC5671554

[7] Z. Xu , L. Wang , S. Dai , et al., “Epidemiologic Trends of and Factors Associated With Overall Survival for Patients With Gastroenteropancreatic Neuroendocrine Tumors in the United States,” JAMA Network Open 4 (2021): e2124750. 34554237 10.1001/jamanetworkopen.2021.24750 PMC8461504

[8] J. Son , I. J. Park , D. H. Yang , et al., “Oncological Outcomes According to the Treatment Modality Based on the Size of Rectal Neuroendocrine Tumors: A Single‐center Retrospective Study,” Surgical Endoscopy 36 (2022): 2445–2455. 34009477 10.1007/s00464-021-08527-6

[9] L. de Mestier , H. Brixi , R. Gincul , et al., “Updating the Management of Patients With Rectal Neuroendocrine Tumors,” Endoscopy 45 (2013): 1039–1046. 24163193 10.1055/s-0033-1344794

[10] H. Inoue , K. Takeshita , H. Hori , et al., “Endoscopic Mucosal Resection With a Cap‐fitted Panendoscope for Esophagus, Stomach, and Colon Mucosal Lesions,” Gastrointestinal Endoscopy 39 (1993): 58–62. 8454147 10.1016/s0016-5107(93)70012-7

[11] Y. Imada‐Shirakata , M. Sakai , T. Kajiyama , et al., “Endoscopic Resection of Rectal Carcinoid Tumors Using Aspiration Lumpectomy,” Endoscopy 29 (1997): 34–38. 9083735 10.1055/s-2007-1024058

[12] A. Ono , T. Fujii , Y. Saito , et al., “Endoscopic Submucosal Resection of Rectal Carcinoid Tumors With a Ligation Device,” Gastrointestinal Endoscopy 57 (2003): 583–587. 12665777 10.1067/mge.2003.142

[13] Y. Mashimo , T. Matsuda , T. Uraoka , et al., “Endoscopic Submucosal Resection With a Ligation Device Is an Effective and Safe Treatment for Carcinoid Tumors in the Lower Rectum,” Journal of Gastroenterology and Hepatology 23 (2008): 218–221. 18289355 10.1111/j.1440-1746.2008.05313.x

[14] Y. J. Kim , S. K. Lee , J. H. Cheon , et al., “Efficacy of Endoscopic Resection for Small Rectal Carcinoid: A Retrospective Study,” Korean Journal of Gastroenterology 51 (2008): 174–180. 18451691

[15] N. Yamaguchi , H. Isomoto , H. Nishiyama , et al., “Endoscopic Submucosal Dissection for Rectal Carcinoid Tumors,” Surgical Endoscopy 24 (2010): 504–508. 19585069 10.1007/s00464-009-0606-0

[16] S. Ito , K. Hotta , A. Matsuda , et al., “Short‐term Outcomes of Endoscopic Resection for Colorectal Neuroendocrine Tumors: Japanese Multicenter Prospective C‐NET Study,” Dig Endosc 35 (2023): 563–571. 10.1111/den.14728 37986226

[17] T. Ito , T. Masui , I. Kitagawa , et al., “JNETS Clinical Practice Guidelines for Gastroenteropancreatic Neuroendocrine Neoplasms: Diagnosis, Treatment, and Follow‐up: A Synopsis,” Journal of Gastroenterology 56 (2021): 1033–1044. 34586495 10.1007/s00535-021-01827-7 PMC8531106

[18] S. Tanaka , H. Kashida , Y. Saito , et al., “JGES Guidelines for Colorectal Endoscopic Submucosal Dissection/Endoscopic Mucosal Resection,” Dig Endosc 27 (2015): 417–434. 25652022 10.1111/den.12456

[19] D. Hihara , Y. Saito , M. Sekiguchi , et al., “Factors Associated With Increased Duration of Endoscopic Submucosal Dissection for Rectal Tumors: A 22‐year Retrospective Analysis,” Gastrointestinal Endoscopy 98 (2023): 420–427. 37061136 10.1016/j.gie.2023.04.005

[20] J. Hayasaka , S. Hoteya , S. Yamashita , et al., “Traction Devices May Not Affect the Vertical Margin Distance in Endoscopic Submucosal Dissection of Rectal Neuroendocrine Tumors,” Cureus 16 (2024): e58976. 38800345 10.7759/cureus.58976 PMC11127712

[21] Y. Saito , T. Uraoka , T. Matsuda , et al., “Risk Factors for Intraoperative Perforation During Endoscopic Submucosal Dissection,” World Journal of Gastroenterology 23, no. 3 (2017): 478–485, 10.3748/wjg.v23.i3.478. 28210084 PMC5291853

[22] M. Esaki , S. Oka , S. Tanaka , et al., “Reduction in the Procedure Time of Hybrid ESD for Early Gastric Neoplasms: A Multicenter Retrospective Study,” Therapeutic Advances in Gastroenterology 13 (2020): 1756284820924202, 10.1177/1756284820924202. PMC7412903 32821288

[23] C. K. Oh , H. H. Chung , J. K. Park , et al., “Comparing Underwater Endoscopic Submucosal Dissection and Conventional Endoscopic Submucosal Dissection for Large Laterally Spreading Tumor: A Randomized Controlled Trial,” Gastrointestinal Endoscopy 99, no. 6 (2024): 827–836.e2, 10.1016/j.gie.2024.04.035. 38969234

[24] A. Matsumoto , S. Tanaka , K. Chayama , et al., “Outcome of Endoscopic Submucosal Dissection for Colorectal Tumors Accompanied by Fibrosis,” Scandinavian Journal of Gastroenterology 45 (2010): 1329–1337. 20626303 10.3109/00365521.2010.495416

[25] M. Higashimaya , S. Oka , K. Chayama , et al., “Outcome of Endoscopic Submucosal Dissection for Gastric Neoplasm in Relationship to Endoscopic Classification of Submucosal Fibrosis,” Gastric Cancer 16 (2013): 404–410. 23053827 10.1007/s10120-012-0203-0

[26] T. Yamashina , H. Ishihara , T. Takasago , et al., “Features of Electrocoagulation Syndrome After Endoscopic Submucosal Dissection for Colorectal Tumors,” Journal of Gastroenterology and Hepatology 30, no. 4 (2015): 750–755, 10.1111/jgh.12791. 26202127

[27] T. Wallenhorst , L. J. Masgnaux , J. Grimaldi , et al., “Obtaining a Free Vertical Margin Is Challenging in Endoscopic Submucosal Dissection of a Rectal Neuroendocrine Tumor: Use of Adaptive Traction to Improve Exposure in a Child,” Endoscopy 55 (2023): E763–E764. 37236253 10.1055/a-2085-0449 PMC10219758

[28] J. Liu and N. Fang , “Traction by Dental Floss Loop for Adequate Submucosal Dissection Depth in a Rectal Neuroendocrine Tumor,” Endoscopy 55 (2023): E326–E327. 36513110 10.1055/a-1974-9297 PMC9833942

[29] R. Ichijima , H. Ikehara , T. Gotoda , et al., “Randomized Controlled Trial Comparing Conventional and Traction Endoscopic Submucosal Dissection for Early Colon Tumor (CONNECT‐C trial),” Digestive Endoscopy 35 (2023): 580–589. 10.1111/den.14426 35997037

[30] M. Ito , et al., “Multicenter Randomized Controlled Trial Comparing Conventional vs Traction‐assisted Endoscopic Submucosal Dissection for Colorectal Tumors,” Endoscopy International Open 11, no. 6 (2023): E525–E532, 10.1055/a-2094-6574.

[31] T. Noguchi , S. Ushimaru , Y. Takeuchi , et al., “Risk Factors for Complications of Colorectal Endoscopic Submucosal Dissection: Retrospective Study With Prospectively Collected Data,” World Journal of Gastroenterology 23, no. 34 (2017): 6224–6231, 10.3748/wjg.v23.i34.6224.

[32] T. Kitagawa , Y. Hayashi , N. Sakamoto , et al., “Endoscopic Submucosal Dissection versus Modified Endoscopic Mucosal Resection for Small Rectal Neuroendocrine Tumors: A Propensity Score–matched Multicenter Study,” Scientific Reports 14 (2024): 82082, 10.1038/s41598-024-82082-6.

[33] E. M. Song , D. H. Yang , J. Kim , et al., “Comparison of Modified Endoscopic Mucosal Resection and Endoscopic Submucosal Dissection for Rectal Neuroendocrine Tumors,” Surgical Endoscopy 30, no. 6 (2016): 2597–2603, 10.1007/s00464-015-4484-z.

[34] P. H. Zhou , X. Wang , Y. Q. Zhang , et al., “Endoscopic Resection for Rectal Neuroendocrine Tumors: A Systematic Review and Meta‐analysis,” World Journal of Gastroenterology 15, no. 6 (2023): 278–288, 10.4253/wjge.v15.i6.278.

[35] B. I. Lee , B. W. Kim , H. K. Kim , et al., “Efficacy of Endoscopic Submucosal Dissection for Rectal Neuroendocrine Tumors and Risk Factors of Incomplete Resection,” Endoscopy 52, no. 7 (2020): 635–639, 10.1055/a-1164-7309.

[36] G. H. Kim , J. I. Kim , B. E. Lee , et al., “Clinical Outcomes of Endoscopic Resection of Small Rectal Neuroendocrine Tumors and Risk Factors of Incomplete Resection,” Gastrointestinal Endoscopy 90, no. 4 (2019): 603–612, 10.1016/j.gie.2019.05.034.

[37] T. Sakamoto , M. Inamori , Y. Sekine , et al., “Feasibility of Endoscopic Submucosal Dissection for Rectal Neuroendocrine Tumors With Submucosal Fibrosis,” Surgical Endoscopy 35, no. 5 (2021): 2094–2102, 10.1007/s00464-020-07507-2.

[38] S. Tanaka , S. Shinozaki , T. Kobayashi , et al., “Comparison of Endoscopic Submucosal Dissection and Modified Endoscopic Mucosal Resection for Rectal Neuroendocrine Tumors: A Multicenter Study,” Scientific Reports 14 (2024): 4281, 10.1038/s41598-024-82082-7. 39948094 PMC11825951

